# Coronary–Bronchial Artery Fistulas: Pathophysiology, Multimodality Imaging, and Contemporary Management

**DOI:** 10.3390/jcdd13060238

**Published:** 2026-05-31

**Authors:** Andrea Falcetta, Francesca Giordana, Paolo Desalvo, Giorgio Baralis, Domenico Vitale, Giuseppe Lauria, Roberta Rossini

**Affiliations:** 1 Cardiology and Intensive Coronary Care Unit Department, Santa Croce e Carle Hospital, 12100 Cuneo, Italy; desalvo.p@ospedale.cuneo.it (P.D.); baralis.g@ospedale.cuneo.it (G.B.); roberta.rossini2@gmail.com (R.R.); 2 Intensive Care Unit Department, Santa Croce e Carle Hospital, 12100 Cuneo, Italy; vitale.d@ospedale.cuneo.it; 3 Emergency Medicine Department, Santa Croce e Carle Hospital, 12100 Cuneo, Italy; lauria.g@ospedale.cuneo.it

**Keywords:** coronary artery fistulas, coronary–bronchial artery fistulas, pathophysiology, diagnosis, treatment

## Abstract

Coronary–bronchial artery fistulas (CBAFs) represent a rare subset of coronary artery fistulas characterised by an abnormal communication between an epicardial coronary artery and the bronchial arterial circulation. Although historically considered incidental findings, the widespread use of multimodality cardiovascular imaging—particularly coronary computed tomography angiography—has led to increasing recognition of these anomalies in contemporary clinical practice. The clinical significance of CBAFs varies widely and depends primarily on fistula size, shunt magnitude, and associated cardiopulmonary conditions. While many small fistulas remain asymptomatic, larger or haemodynamically significant lesions may result in myocardial ischaemia due to coronary steal, ventricular remodelling, pulmonary manifestations such as haemoptysis, and aneurysmal degeneration of the fistulous tract. A comprehensive evaluation typically requires an integrated multimodality approach combining anatomical imaging, functional ischaemia testing, and, in selected cases, invasive haemodynamic assessment. Management strategies range from conservative surveillance in small asymptomatic fistulas to percutaneous or surgical closure in symptomatic or haemodynamically significant lesions. This review provides an updated overview of the epidemiology, pathophysiology, diagnostic evaluation, and management of CBAFs. Particular emphasis is placed on size-based clinical stratification, multimodality imaging strategies, and contemporary therapeutic approaches, with the aim of offering a practical framework for the diagnosis and longitudinal management of patients with this uncommon but clinically relevant coronary anomaly.

## 1. Introduction

Coronary artery anomalies are uncommon findings, although their reported prevalence varies according to the population studied, the diagnostic modality used, and the anatomical classification adopted. Older autopsy series have reported a prevalence of approximately 0.3%, whereas angiographic studies have described prevalences of up to 5.64%; however, these figures refer to coronary artery anomalies as a whole rather than specifically to coronary artery fistulas (CAFs). Within this broader spectrum, CAFs represent an uncommon but potentially clinically relevant subgroup of coronary vascular anomalies [1,2,3,4].

Among CAFs, coronary–bronchial artery fistulas (CBAFs) represent a particularly rare and probably under-recognised phenotype, characterised by an abnormal communication between an epicardial coronary artery and the bronchial arterial circulation. Historically regarded as incidental angiographic findings of limited clinical relevance, these lesions have gained increasing attention in recent years, largely owing to advances in multimodality cardiovascular imaging, particularly coronary computed tomography angiography (CCTA), which has improved their detection and anatomical characterisation [5,6,7].

**Figure 1:**
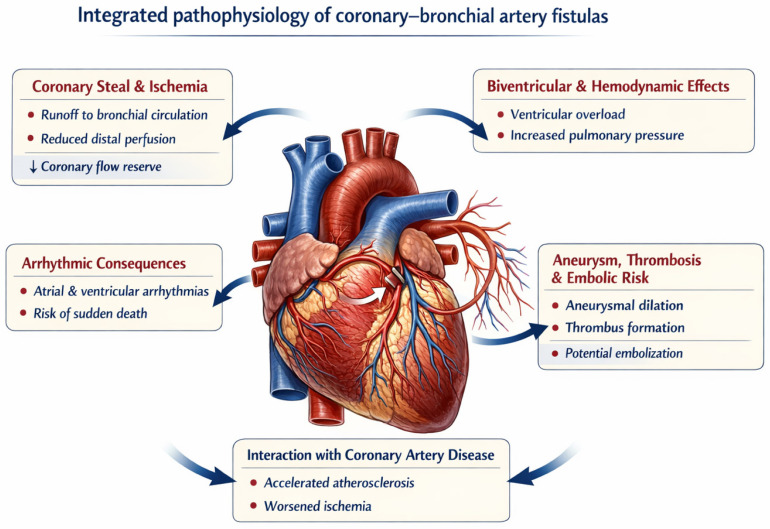
Pathophysiological framework of coronary–bronchial artery fistulas. The figure summarises coronary steal physiology, bronchial hyperperfusion, chamber remodelling, arrhythmic consequences, and aneurysmal/thrombotic complications, together with the potential interaction between CBAFs and coexisting coronary artery disease.

**Figure 2:**
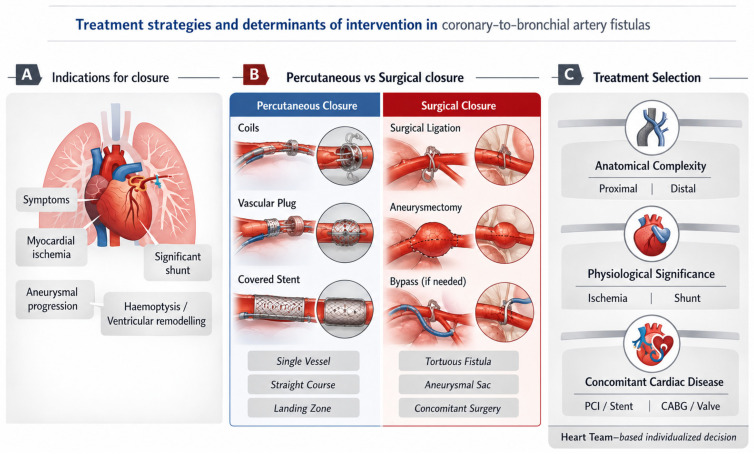
Contemporary treatment strategies for coronary–bronchial artery fistulas. Percutaneous and surgical approaches are illustrated together with the principal determinants of treatment selection, including anatomical suitability, physiological significance, fistula size, and the presence of concomitant coronary or surgical disease. In the left panel are indicated the determinants for closure; in the central panel, the percutaneous and surgical treatments, along with the characteristics in favor of each treatment; and in the right panel, the concurrent anatomical, physiological, and cardiac disease(s) required to consider a percutaneous rather than a surgical treatment. Abbreviations: CABG = coronary artery bypass grafting; PCI = percutaneous coronary intervention.

**Figure 3:**
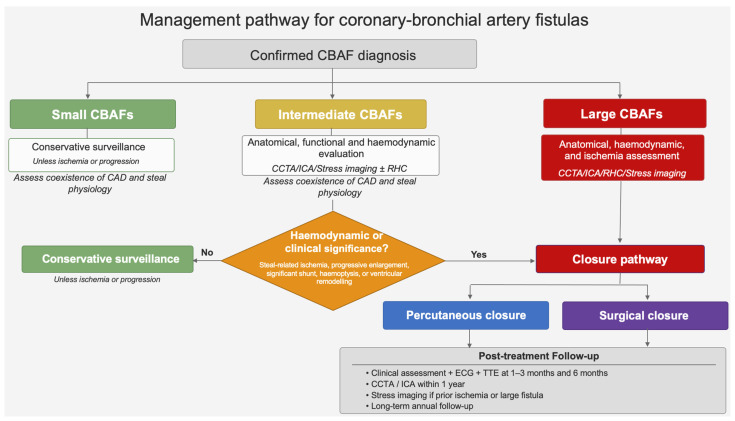
Practical flow chart for management and surveillance of coronary–bronchial artery fistulas. The algorithm integrates fistula size, symptoms, ischaemia testing, haemodynamic assessment, and anatomical complexity to guide surveillance, closure, and choice of therapeutic strategy. Abbreviations: CAD = coronary artery disease; CBAFs = coronary–bronchial artery fistulas; CCTA = coronary computed tomography angiography; ECG = electrocardiogram; ICA = invasive coronary angiography; RHC = right heart catheterization; TTE = transthoracic echocardiography.

Although many CBAFs remain clinically silent, selected patients may develop clinically meaningful consequences related to abnormal coronary flow, myocardial ischaemia, chamber remodelling, pulmonary vascular complications, or aneurysmal degeneration. The pathophysiological, diagnostic, and therapeutic implications of these lesions are summarised in the Graphical Abstract and further detailed in Figure 1, Figure 2 and Figure 3 [8,9,10,11,12,13].

## 2. Epidemiology and Prevalence

CBAFs represent a rare and probably under-recognised subtype of CAFs. Their true prevalence remains difficult to establish, largely because many lesions are clinically silent and because the available evidence is derived predominantly from retrospective angiographic series, computed tomography datasets, and case-based reports rather than dedicated population-based studies. Accordingly, published epidemiological estimates should be interpreted with caution [5,7,13].

More broadly, the reported prevalence of CAFs varies substantially according to the diagnostic modality, referral setting, and anatomical classification used. Invasive angiographic series have historically identified CAFs as uncommon findings, with reported prevalences generally ranging from 0.2% to 0.9%, although wider ranges have been described in selected populations. By contrast, older autopsy data reporting a prevalence of approximately 0.3% refer to coronary artery anomalies as a whole rather than specifically to CAFs and should therefore not be directly extrapolated to CBAFs. In contemporary practice, the increasing use of CCTA has significantly improved the detection of small, tortuous, and extracardiac vascular communications, thereby reshaping the apparent epidemiology of these lesions [1,2,3,4,5,11,13,14,15].

Within the broader CAFs spectrum, CBAFs appear to account for only a small minority of cases, with published estimates of approximately 0.4–0.6%. However, these figures remain inherently imprecise because many reports aggregate anatomically distinct fistula phenotypes under the generic CAFs label, while dedicated CBAFs series remain limited in size [16,17].

Thus, the growing recognition of CBAFs in recent years most likely reflects improved ascertainment rather than a true increase in incidence. Despite their rarity, these fistulas are clinically relevant because their presentation may range from incidental and haemodynamically insignificant lesions to anatomically complex or physiologically significant forms associated with myocardial ischaemia, haemoptysis, chamber remodelling, or aneurysmal degeneration [5,6,8,17,18].

## 3. Clinical Presentation

The clinical presentation of CBAFs is highly variable and depends primarily on the size of the fistulous connection, the magnitude of shunt flow, and the presence of concomitant cardiovascular or pulmonary disease. In many patients—particularly those with small fistulas—CBAFs are detected incidentally during coronary imaging or thoracic computed tomography performed for unrelated indications [6,11,13].

When symptoms occur, they most commonly reflect myocardial ischaemia resulting from the coronary steal phenomenon. In this setting, blood flow is diverted from the distal myocardial circulation into the bronchial arterial network, which may represent a relatively lower-resistance pathway. During conditions of increased myocardial oxygen demand, such as physical exertion or tachycardia, this diversion of flow may lead to relative hypoperfusion of the myocardial territory supplied by the feeding coronary artery, manifesting clinically as exertional angina or dyspnoea [8,11].

Pulmonary complications represent another characteristic component of the clinical spectrum. Increased flow through the bronchial arterial circulation may result in vascular dilation and fragility, occasionally leading to haemoptysis or recurrent pulmonary infections. Such complications appear more frequently in patients with underlying pulmonary disease, including bronchiectasis or chronic inflammatory lung disorders, which may further augment bronchial vascular flow and amplify the haemodynamic impact of the fistula [6,17,18].

Less frequently, CBAFs may be associated with arrhythmias, progressive ventricular remodelling with systolic dysfunction, or aneurysmal dilation of the fistulous tract, particularly in long-standing or high-flow lesions. Rare complications described in the literature include infective endocarditis/endarteritis, myocardial infarction, and sudden cardiac death, although these events remain uncommon in small, haemodynamically insignificant lesions [10,18,19,20,21].

## 4. Pathophysiology

### 4.1. General Pathophysiological Mechanisms

From a pathophysiological perspective, CBAFs may be either congenital or acquired. Congenital forms are generally attributed to persistence of primitive embryologic vascular connections between the developing coronary circulation and the bronchial arterial plexus. During normal cardiac development, primitive vascular channels and myocardial sinusoids regress as the mature coronary circulation forms; failure of this involution may result in persistent anomalous communications that later manifest as coronary fistulas. Acquired forms are more frequently associated with chronic pulmonary or inflammatory conditions, such as bronchiectasis, chronic infection, or longstanding pulmonary inflammation. In these settings, chronic hypoxia, angiogenic signalling, and vascular remodelling may enlarge pre-existing microvascular anastomoses or promote the development of new collateral channels between the coronary and bronchial circulations [11,17].

The clinical expression of CBAFs is determined not only by fistula size, but also by the anatomical and haemodynamic properties of the recipient vascular bed. In contrast to classical coronary-cameral fistulas, which usually drain into low-pressure right-sided cardiac chambers, CBAFs communicate with the bronchial circulation, a systemic arterial network characterised by complex downstream venous drainage. Bronchial arteries are connected to bronchial veins, which drain partly into the azygos and hemiazygos venous systems and partly into the pulmonary veins. This peculiar vascular arrangement is clinically relevant because it explains why the haemodynamic consequences of CBAFs may not be limited to a simple arterial runoff phenomenon, but may instead involve both right- and left-sided cardiopulmonary loading conditions. It also provides an important rationale for invasive haemodynamic assessment with right heart catheterization (RHC) in selected patients, particularly when the degree of shunting, pulmonary pressure burden, or chamber remodelling remains uncertain after non-invasive imaging [6,11,17,18].

### 4.2. Coronary Steal and Myocardial Ischaemia

One of the principal mechanisms underlying the clinical manifestations of CBAFs is the coronary steal phenomenon. The fistulous communication creates a continuous runoff pathway from the epicardial coronary artery toward the bronchial vascular bed, thereby reducing effective antegrade flow to the distal myocardial territory supplied by the donor vessel. Under resting conditions, coronary autoregulation may compensate for moderate flow diversion. However, during exercise, tachycardia, or other states of increased myocardial oxygen demand, the fixed low-resistance runoff through the fistula may impair the normal increase in coronary flow reserve, resulting in relative myocardial hypoperfusion [8,11,18].

The severity of coronary steal depends on several interacting factors, including fistula size, the pressure gradient across the communication, distal resistance within the bronchial vascular bed, and the presence of concomitant coronary artery disease (CAD). This mechanism explains why patients may present with exertional angina, dyspnoea, or silent ischaemia documented by functional testing. It also explains why stress perfusion imaging plays a central role in identifying physiologically significant fistulas, particularly when anatomical size alone is insufficient to predict clinical relevance [8,11,18].

### 4.3. Biventricular and Haemodynamic Consequences

Although CBAFs are often conceptually described as producing a “left-to-left” shunt, their physiological consequences may be more complex because of the mixed venous drainage of the bronchial circulation. The fraction of bronchial venous return draining into the pulmonary veins contributes to left-sided volume loading, which may favour progressive left atrial and left ventricular enlargement over time. At the same time, drainage through the azygos and hemiazygos systems ultimately returns to the right heart, potentially contributing to right-sided volume burden and pulmonary circulatory consequences in selected high-flow CBAFs [6,11,18].

Accordingly, intermediate or large CBAFs may produce progressive chamber remodelling that is not exclusively left-sided and may, in some patients, involve both ventricles. Repetitive ischaemia due to coronary steal may further aggravate myocardial dysfunction by promoting fibrosis, microvascular dysfunction, and impaired contractile reserve. If sustained over time, the combined effects of chronic volume load and ischaemic injury may ultimately lead to symptomatic heart failure. This pathophysiological framework is clinically important because it explains why transthoracic echocardiography may reveal biatrial or biventricular remodelling, and why RHC may be useful in selected cases to quantify shunt burden, measure pulmonary artery pressures, and define the overall haemodynamic significance of the lesion [22,23,24,25].

### 4.4. Arrhythmic Consequences

Chronic ischaemia, atrial or ventricular dilatation, and replacement or interstitial fibrosis may create an arrhythmogenic substrate in patients with CBAFs. Atrial fibrillation may occur in association with atrial enlargement and chronic volume stress, whereas ventricular arrhythmias are more likely in the presence of ischaemia, scar formation, or advanced ventricular remodelling. Accordingly, arrhythmias should be viewed as downstream manifestations of the chronic haemodynamic and ischaemic burden imposed by the fistula [8,10,20].

### 4.5. Aneurysmal Degeneration, Thrombosis, and Embolic Risk

Longstanding abnormal flow may induce progressive dilation of both the feeding coronary artery and the fistulous tract itself. Chronic wall stress and turbulent flow may promote aneurysmal degeneration, particularly in larger or more tortuous fistulas. Once aneurysmal enlargement develops, sluggish flow and areas of relative stasis may predispose to thrombus formation within the dilated coronary segment or the fistulous channel [6,12,18,25].

This mechanism is clinically relevant because thrombotic material may embolize distally toward the coronary bed, causing myocardial infarction or microvascular ischaemia. In rare cases, embolization toward the bronchial or pulmonary circulation may also occur. Rupture of a fistulous aneurysm is distinctly uncommon, but represents one of the most catastrophic complications, potentially leading to haemopericardium, massive haemoptysis, or sudden cardiovascular collapse [6,12,18,25].

### 4.6. Interaction with Coexisting Coronary Artery Disease

In adult patients, the coexistence of obstructive CAD adds further complexity to the pathophysiology of CBAFs. In this setting, myocardial ischaemia may result from the combined effects of fixed epicardial stenosis and dynamic steal physiology. Flow diversion through the fistula may exacerbate ischaemia in territories already compromised by reduced coronary flow reserve, particularly during stress. Conversely, severe proximal or mid-vessel stenosis may reduce fistula flow and partially mask its haemodynamic relevance [18,26,27,28].

This interaction explains why anatomical assessment alone is often insufficient and why an integrated physiological approach is required. The relative contribution of CAD and fistulous steal to symptoms or inducible ischaemia should therefore be assessed using multimodality perfusion imaging and, when appropriate, invasive coronary physiology [26,27,28].

### 4.7. Clinical Implications

Taken together, these mechanisms indicate that the pathophysiological consequences of CBAFs extend beyond a simple anomalous vascular communication. Rather, they reflect a complex interplay among coronary runoff, bronchial vascular resistance, mixed systemic and pulmonary venous drainage, myocardial perfusion, and progressive cardiopulmonary remodelling. This pathophysiological complexity underlies the heterogeneity of clinical presentation, the potential for biventricular involvement, and the selective role of RHC in patients with intermediate or large fistulas, unexplained chamber enlargement, suspected haemodynamically significant shunting, or pulmonary pressure abnormalities. Accordingly, accurate clinical assessment requires an integrated diagnostic approach combining anatomical definition, physiological evaluation, and haemodynamic characterisation, as summarised in Figure 1 [11,22,23,24].

## 5. Diagnostic Evaluation

Accurate diagnosis and risk stratification of CBAFs rely on a multimodality imaging strategy integrating anatomical delineation, functional ischaemia testing, and, in selected cases, invasive haemodynamic assessment. The complementary strengths and limitations of the principal techniques are summarised in Table 1, whereas their integration into the overall clinical pathway is outlined in Figure 3 [22,29,30,31,32,33].

### 5.1. Coronary Computed Tomography Angiography

CCTA currently represents the imaging modality of choice for the non-invasive anatomical characterisation of CBAFs. Owing to its high spatial resolution and isotropic voxel acquisition, CCTA allows precise delineation of the fistulous origin, course, termination, and potential aneurysmal dilatation, while simultaneously assessing the presence of concomitant CAD [15,29,32,34,35,36,37,38].

Beyond simple anatomical depiction, CCTA provides three-dimensional volumetric reconstruction and multiplanar reformatting, facilitating comprehensive evaluation of the spatial relationship between the fistula and adjacent mediastinal, pulmonary, and vascular structures. This is particularly relevant in CBAFs, where communication with bronchial arterial branches may be tortuous and anatomically complex. In addition, contemporary CT scanners allow assessment of shunt-related chamber enlargement and indirect signs of haemodynamic relevance [29,30,34,35,36,37].

Importantly, CCTA enables integrated evaluation of CAD, which is frequently detected incidentally in this patient population. In selected cases, fractional flow reserve derived from CT (FFR-CT) may provide incremental information regarding the functional significance of epicardial stenoses, helping to distinguish ischaemia attributable to coronary steal from that related to obstructive atherosclerotic disease [28,29,39,40].

Given its diagnostic accuracy, reproducibility, and ability to guide therapeutic planning (percutaneous versus surgical closure), CCTA is generally considered first-line imaging in stable patients with suspected or incidentally detected CBAFs [28,29,30,31,34,35,36,37,41].

### 5.2. Invasive Coronary Angiography

Invasive coronary angiography (ICA) remains the historical gold standard for the diagnosis of coronary fistulas and continues to play a pivotal role when intervention is contemplated. ICA provides dynamic real-time visualisation of coronary flow patterns and permits selective opacification of the fistulous tract, allowing assessment of origin, drainage site, and competitive coronary flow [11,18,25,32,42,43].

However, ICA may underestimate the full three-dimensional complexity of CBAFs due to vessel overlap and limited projection angles. In this context, CCTA frequently offers superior anatomical definition. Nonetheless, ICA remains indispensable when transcatheter closure is planned, as it enables simultaneous diagnostic confirmation and therapeutic intervention [11,29,30,42].

Physiological assessment may be incorporated during ICA. Fractional flow reserve (FFR) or instantaneous wave-free ratio (iFR) can help differentiate ischaemia secondary to obstructive CAD from steal phenomena related to the fistula. In selected cases with suspected haemodynamically significant shunting or unexplained right-sided chamber loading or pulmonary pressure abnormalities, RHC performed during the same procedure allows calculation of the Qp:Qs ratio and pulmonary vascular resistance, providing haemodynamic quantification that may influence management [24,26,27].

Therefore, ICA remains an essential component of the diagnostic pathway in patients in whom non-invasive imaging suggests haemodynamically significant shunting, when symptoms remain unexplained, or when transcatheter closure is being considered [11,24,42,43].

### 5.3. Echocardiography

Transthoracic echocardiography (TTE) represents the initial imaging modality in most patients with suspected structural heart disease and plays an important complementary role in CBAFs. Although direct visualisation of the fistulous tract is uncommon in adults, echocardiography provides crucial information regarding cardiac chamber dimensions, ventricular systolic and diastolic function, valvular abnormalities, and indirect signs of volume overload [22,23,30,32,44].

Colour Doppler imaging may occasionally demonstrate abnormal continuous flow patterns, particularly in cases with sizable shunting. More frequently, echocardiography identifies secondary consequences such as ventricular enlargement, pulmonary hypertension, or regional wall motion abnormalities suggestive of ischaemia [22,23,30].

Transesophageal echocardiography (TEE) may improve visualisation in selected patients, particularly when the drainage site is proximal or when differential diagnosis with other vascular malformations is required. Intra-procedural TEE can also assist during surgical or percutaneous closure [18,23,30].

Importantly, echocardiography is central to longitudinal surveillance. Serial assessment allows detection of progressive chamber enlargement, development of pulmonary hypertension, or ventricular dysfunction, which may indicate increasing haemodynamic significance and prompt reconsideration of intervention [22,23,30,44].

### 5.4. Myocardial Perfusion Imaging (SPECT/PET)

Functional ischaemia testing represents a key component of the evaluation of CBAFs, particularly in patients with intermediate-sized fistulas in whom anatomical size alone does not reliably predict physiological significance. Stress perfusion imaging modalities—including myocardial perfusion SPECT and PET—allow detection of inducible perfusion defects related to coronary steal within the myocardial territory supplied by the feeding coronary artery. Demonstration of reversible ischaemia strongly supports consideration of fistula closure irrespective of the absolute anatomical diameter. In addition, functional imaging provides a useful reference for post-intervention assessment and longitudinal surveillance [11,28,30,31,32].

### 5.5. Cardiac Magnetic Resonance

Cardiac magnetic resonance (CMR), including stress CMR, provides a comprehensive, radiation-free, multiparametric evaluation of CBAFs, integrating anatomical, functional, and tissue characterisation data within a single examination. While CCTA remains the reference modality for high-resolution anatomical mapping of the fistulous tract, CMR may provide incremental value in assessing physiological relevance and myocardial consequences [18,30,31,32,45].

Contrast-enhanced MR angiography may delineate the proximal origin and aneurysmal segments of larger fistulas; however, the principal strength of CMR lies in functional assessment. Phase-contrast velocity mapping can support semi-quantitative estimation of abnormal flow and contribute to shunt evaluation when invasive assessment is deferred. Cine imaging provides gold standard quantification of biventricular volumes and function, enabling early detection of remodelling related to chronic volume overload [18,30,45].

CMR may be particularly valuable in intermediate-sized CBAFs, allowing detection of inducible ischaemia attributable to coronary steal. In addition, late gadolinium enhancement (LGE) identifies myocardial fibrosis or prior silent infarction, refining risk stratification and therapeutic timing. Quantitative perfusion approaches, when available, may further enhance detection of subtle perfusion abnormalities [18,30,31,45].

CMR is especially valuable in younger patients requiring radiation minimization, in cases with inconclusive nuclear imaging, and in post-treatment surveillance when residual ischaemia or ventricular remodelling is suspected. Accordingly, CMR should be considered a complementary second-line modality within a multimodality imaging strategy for CBAFs, particularly when functional significance rather than purely anatomical definition is the clinical priority [18,30,31,45].

### 5.6. Right Heart Catheterization

RHC remains the gold standard for quantitative assessment of shunt magnitude through calculation of the pulmonary-to-systemic flow ratio (Qp:Qs), as well as for measurement of pulmonary artery pressures and pulmonary vascular resistance. Although not routinely required in every patient, RHC is particularly useful when the haemodynamic significance of a fistula remains uncertain after non-invasive imaging. In patients with intermediate or large CBAFs, measurement of the Qp:Qs ratio provides objective quantification of shunt burden, with values ≥ 1.5 commonly considered indicative of haemodynamically significant shunting that may support consideration of closure. RHC is also valuable prior to intervention to establish baseline pulmonary pressures and guide procedural planning [24,25].

Taken together, these imaging modalities provide complementary information, and no single technique is sufficient in all clinical scenarios. Anatomical definition, physiological relevance, and haemodynamic burden should therefore be assessed within an integrated multimodality framework tailored to fistula size, symptoms, and the suspected mechanism of clinical significance. The principal strengths and limitations of each multimodality evaluation are summarised in Table 1 [22,24,29,31,32,33].

## 6. Clinical Classification and Management of CBAFs

CBAFs may be classified according to size and potential clinical relevance using two complementary approaches. The most widely used classification in clinical practice is based on the absolute diameter of the fistulous vessel, typically categorising lesions as small (≤2.5 mm), intermediate (2.6–3.9 mm), and large (≥4.0 mm). This pragmatic approach is commonly adopted in observational studies and clinical reports because it can be readily applied using ICA or CCTA and correlates reasonably well with the likelihood of haemodynamic significance, myocardial ischaemia due to coronary steal, and aneurysmal degeneration of the fistulous tract [10,18,19,25,42,43].

An alternative classification considers the diameter of the fistula relative to the native coronary artery, defining small fistulas as those measuring less than the reference coronary vessel diameter, intermediate fistulas as those measuring between one and two times the diameter of the native coronary artery, and large fistulas as those exceeding twice the diameter of the feeding coronary artery. This relative approach may better reflect the proportion of coronary blood flow diverted through the fistulous pathway and therefore provides a potentially more physiologically meaningful estimate of shunt severity. However, it is less consistently used in routine clinical practice because of measurement variability and the absence of universally standardised thresholds [11,25,33].

The management of CBAFs requires an individualised approach integrating anatomy, haemodynamic significance, symptoms, and associated cardiopulmonary disease. Because these anomalies are frequently detected incidentally during coronary imaging, therapeutic decisions must balance the often benign natural history of small lesions against the potential risk of complications associated with larger or physiologically significant fistulas. A practical size-based framework for management and surveillance is summarised in Table 2. Because of the rarity of CBAFs and the absence of randomised clinical trials, most management recommendations are derived from observational studies, extrapolation from the broader coronary artery fistula literature, and expert consensus [9,10,18,19,20,33].

Small CBAFs are typically haemodynamically insignificant and are often discovered incidentally during coronary imaging. In the absence of symptoms, inducible ischaemia, or progressive enlargement, conservative management with periodic clinical and imaging follow-up is generally appropriate. Observational series suggest that most small fistulas remain stable over time and are associated with a very low risk of complications [9,10,20].

A clinically relevant scenario frequently encountered in adult patients is the coexistence of a small CBAF and obstructive CAD. In such cases, myocardial ischaemia may arise either from epicardial coronary stenosis or from coronary steal physiology related to the fistula. Current evidence suggests that routine closure of small CBAFs solely because of concomitant CAD is not justified in the absence of demonstrable fistula-related ischaemia. Instead, a physiology-guided approach is recommended [26,27,28,31,33].

Functional imaging techniques—such as myocardial perfusion SPECT, PET, or stress CMR—play a key role in this context because they allow accurate localization of the ischaemic territory and differentiation between ischaemia caused by epicardial coronary stenosis and steal-related perfusion abnormalities. When ischaemia is attributable exclusively to obstructive CAD, coronary revascularisation alone (either percutaneous coronary intervention or coronary artery bypass grafting) is generally sufficient [26,27,28,30,31].

Conversely, combined treatment including both coronary revascularisation and fistula closure may be considered when residual ischaemia persists after adequate revascularisation, when steal physiology is clearly demonstrated in the myocardial territory supplied by the feeding coronary artery, or when progressive dilation or aneurysmal transformation of the fistulous tract is observed [20,21,31,33].

For these reasons, the management of small CBAFs in the presence of CAD should be primarily physiology-driven rather than anatomy-driven, emphasising careful functional assessment before considering interventional closure [26,27,28,33].

Intermediate-sized CBAFs represent the most heterogeneous and clinically debated subgroup, as their haemodynamic significance cannot be reliably predicted by anatomical size alone. In this setting, management decisions should be guided by an integrated assessment combining anatomical imaging, functional ischaemia testing, and haemodynamic evaluation [11,19,33].

Multimodality imaging plays a central role in the evaluation of these fistulas. CCTA provides detailed characterisation of fistula origin, course, drainage site, and aneurysmal change or progressive dilatation. ICA remains essential when interventional treatment is contemplated and allows simultaneous physiological assessment when needed. Functional testing is particularly important in this subgroup because intermediate fistulas may produce clinically relevant coronary steal even in the absence of overt structural abnormalities. Stress perfusion imaging—including SPECT, PET, or stress CMR—should therefore be considered to detect inducible ischaemia in the myocardial territory supplied by the feeding coronary artery. Demonstration of reversible perfusion defects strongly supports a strategy of fistula closure irrespective of the absolute anatomical diameter. RHC may provide additional haemodynamic information in selected patients, particularly when there is evidence of chamber enlargement, unexplained dyspnoea, or discordance between symptoms and imaging findings. Quantification of the shunt ratio (Qp:Qs) allows objective assessment of the haemodynamic burden, and a value ≥ 1.5 is commonly regarded as indicative of a haemodynamically significant shunt that may justify intervention [29,30,31,33].

Accordingly, management of intermediate CBAFs should remain individualised and physiology-guided. Closure may be considered in the presence of symptoms, demonstrable ischaemia, significant shunt physiology, recurrent haemoptysis, progressive enlargement, or ventricular remodelling, whereas conservative surveillance remains appropriate in asymptomatic patients without evidence of physiological or structural progression [11,24,25,31,33].

Large CBAFs are generally haemodynamically significant and are associated with a substantially higher risk of complications, including myocardial ischaemia due to coronary steal, ventricular remodelling, bronchial hyperperfusion with haemoptysis, and aneurysmal degeneration of the fistulous tract. Consequently, closure is usually recommended once the diagnosis is established and anatomical feasibility has been defined [9,11,19,20,21].

Multimodality evaluation is essential before intervention. CCTA provides high-resolution anatomical delineation of the fistulous pathway, enabling identification of aneurysmal segments and assessment of the relationship between the fistula and adjacent mediastinal structures. ICA remains the reference technique for procedural planning and allows simultaneous evaluation of concomitant CAD. Functional imaging (stress SPECT, PET, or stress CMR) may document the presence and extent of ischaemia related to coronary steal. RHC may quantify shunt magnitude and measure pulmonary artery pressures. A Qp:Qs ratio ≥ 1.5 or evidence of high-output physiology further supports the indication for definitive closure [22,29,30,31,44].

Overall, early recognition of large CBAFs and timely intervention are crucial to reduce the risk of progressive remodelling and major complications [9,21,31,33].

## 7. Treatment and Outcomes

The therapeutic management of CBAFs should be individualised according to anatomical complexity, haemodynamic significance, ischaemic burden, and associated cardiovascular or pulmonary comorbidities. In contemporary practice, available options include percutaneous closure, surgical repair, and conservative medical management, with the choice of strategy determined primarily by anatomical suitability and physiological impact rather than by size alone. The principal determinants of treatment selection are illustrated in Figure 2, whereas the practical size-based framework is summarised in Table 2 [21,31,33].

### 7.1. Percutaneous Closure

Transcatheter closure has emerged as the preferred first-line therapeutic approach for most anatomically suitable CBAFs, owing to its minimally invasive nature and high procedural success rates in experienced centres. Ideal candidates for percutaneous closure are patients with a single feeding vessel, a relatively straight fistulous course, and a clearly identifiable drainage site allowing stable catheter positioning [5,6,21,31,35,36,37,46,47].

Several occlusion devices may be used depending on fistula morphology and size. Detachable coils or microcoils are commonly employed for small or moderately sized fistulas with a narrow neck, allowing controlled deployment and progressive thrombosis of the fistulous tract. Vascular plugs, such as the Amplatzer vascular plug, are frequently used for larger fistulas with a suitable landing zone, providing more rapid and durable occlusion. In selected cases in which the fistula originates close to a major coronary branch, covered stents may be deployed within the parent coronary artery to exclude the fistulous origin while preserving distal coronary perfusion [21,31,36,37,46,47,48].

Pre-procedural planning relies heavily on multimodality imaging—particularly CCTA and ICA—to delineate the fistulous anatomy and guide device selection. When appropriately selected, transcatheter closure achieves technical success rates exceeding 80–90%, with significant improvement in symptoms and reduction in ischaemic burden [21,29,35,46,47,48,49,50].

### 7.2. Surgical Closure

Surgical repair remains an important therapeutic option in patients with complex fistulous anatomy or contraindications to transcatheter closure. Indications typically include large or tortuous fistulas not amenable to catheter-based techniques, multiple or plexiform fistulous networks, large aneurysmal sacs, or situations in which concomitant cardiac surgery is required [31,46,47,51,52,53,54].

The surgical technique depends on the anatomical configuration of the fistula. In most cases, closure is achieved by direct ligation of the fistulous tract at its origin or drainage site. In the presence of aneurysmal dilation, surgical resection or aneurysmectomy may be required. Cardiopulmonary bypass is used when the fistula involves deep coronary segments or when intracardiac repair is necessary [30,31,46,47,52,53].

Surgical closure provides durable results with reported long-term success rates exceeding 90%, although it is associated with a longer recovery period and slightly higher procedural morbidity compared with transcatheter techniques [31,46,51,52,53].

### 7.3. Determinants of Treatment Strategy

The choice between percutaneous and surgical treatment is primarily determined by anatomical complexity, fistula origin, and the presence of associated cardiovascular disease. Fistulas arising from proximal coronary segments are generally more suitable for transcatheter closure due to easier catheter access and more favourable landing zones for occlusion devices. Conversely, distal fistulas or those associated with aneurysmal dilation of the feeding coronary artery may present technical challenges and may be better managed surgically in selected cases [21,29,30,31,33].

An additional consideration is the coexistence of CAD or other structural cardiac conditions requiring intervention. In patients undergoing surgical revascularisation (CABG) or valve surgery, simultaneous surgical ligation of the fistula is typically performed. Similarly, when percutaneous coronary intervention (PCI) is indicated for obstructive CAD, transcatheter closure of the fistula may be performed during the same procedure when technically feasible. This combined strategy aims to optimise myocardial perfusion by addressing both epicardial stenosis and coronary steal physiology [21,28,30,31].

In contemporary practice, the choice between transcatheter and surgical closure should be individualised within a multidisciplinary Heart Team framework integrating anatomical complexity, physiological significance, and concomitant cardiovascular disease [21,30,31,33].

### 7.4. Post-Closure Haemodynamic Changes and Thrombotic Risk

A recognised phenomenon following closure of moderate or large coronary fistulas is the development of stagnant flow within the dilated feeding coronary artery, particularly when the proximal coronary segment has undergone chronic aneurysmal enlargement. Elimination of the low-resistance runoff pathway may lead to reduced coronary flow velocity and increase the risk of thrombosis within ectatic coronary arteries [12,25,31,51].

This complication is most frequently observed in patients with large distal fistulas associated with markedly dilated coronary arteries, where thrombosis of the proximal vessel may result in acute myocardial infarction or distal embolization. For this reason, careful procedural planning and post-procedural antithrombotic therapy are recommended, particularly in cases with significant coronary ectasia [12,25,31,51].

### 7.5. Clinical Outcomes After Fistula Closure

Both percutaneous and surgical closure of CBAFs are associated with favourable clinical outcomes when appropriately indicated. Successful closure typically results in resolution of coronary steal physiology, improvement or disappearance of ischaemic symptoms, and reduction in pulmonary manifestations such as haemoptysis. In patients with pre-existing ventricular remodelling, early intervention may also lead to stabilisation or partial reversal of ventricular dilation and improvement in functional status [20,21,31,46,53].

Long-term follow-up studies report high rates of durable fistula occlusion and low recurrence rates, particularly when closure is achieved using modern interventional devices or surgical techniques. However, residual shunting or late recanalization may occur in a minority of patients, underscoring the importance of structured imaging surveillance following treatment [20,31,46,53].

Overall, contemporary evidence suggests that both percutaneous and surgical approaches provide excellent long-term outcomes when patients are carefully selected using a multidisciplinary Heart Team approach integrating anatomical, functional, and haemodynamic data [20,21,30,31,35,46,47,51,52,53].

### 7.6. Medical Management

Medical therapy plays primarily a supportive role in the management of CBAFs and does not eliminate the underlying fistulous communication. Pharmacological treatment is therefore generally reserved for patients awaiting definitive closure, for those considered unsuitable for percutaneous or surgical intervention, or as adjunctive therapy following closure procedures [21,31].

Antithrombotic therapy should be individualised according to the therapeutic strategy and the anatomical characteristics of the fistula. Following transcatheter closure, short-term dual antiplatelet therapy is commonly prescribed to reduce the risk of device-related thrombosis, typically followed by long-term single antiplatelet therapy. In patients undergoing surgical ligation of the fistula, single antiplatelet therapy is generally considered sufficient [21,31].

Oral anticoagulation may be indicated in selected clinical scenarios, including the presence of atrial arrhythmias, large aneurysmal coronary segments with stagnant flow, or documented intracoronary or intracavitary thrombus formation, and should follow established guideline-based indications for thromboembolic risk management [21,31,51].

Antianginal therapy—including β-blockers, nitrates, or calcium-channel blockers—may help alleviate ischaemic symptoms related to coronary steal in patients awaiting intervention or in those deemed unsuitable for definitive closure. In patients with ventricular dysfunction or heart failure related to large fistulas, guideline-directed medical therapy—including ACE inhibitors, angiotensin receptor blockers, angiotensin receptor–neprilysin inhibitors, and diuretics—should be instituted according to contemporary heart failure recommendations [28].

Beyond therapeutic management, long-term clinical outcomes in patients with CBAFs are closely related to fistula size, haemodynamic significance, and the adequacy of treatment when intervention is indicated [9,20,21,31].

## 8. Long-Term Surveillance of CBAFs

Although prospective surveillance protocols are lacking, long-term follow-up remains an essential component of CBAF management, both in conservatively treated patients and after definitive closure. The aims of follow-up are to confirm durable occlusion, detect late complications, and identify interval progression in conservatively managed patients. A practical follow-up algorithm is presented in Figure 3, and a size-based surveillance framework is summarised in Table 2 [23,24,30,31,33].

In patients with small CBAFs who remain asymptomatic and show no evidence of ventricular remodelling or inducible ischaemia, a relatively conservative follow-up strategy is appropriate. Clinical reassessment may be considered every 12–24 months, focusing on the development of exertional symptoms, arrhythmias, or pulmonary manifestations such as haemoptysis. Structural evaluation with TTE may be performed approximately every 2–3 years to monitor ventricular size, systolic function, and pulmonary pressures, while repeat anatomical imaging with CCTA is generally reasonable every 3–5 years to exclude progressive dilation or aneurysmal transformation of the fistulous tract. Routine functional ischaemia testing is not required in stable patients; however, stress imaging with SPECT, PET, or stress CMR should be promptly performed if symptoms develop or if structural imaging suggests possible progression. RHC is generally unnecessary in this subgroup but may be considered when non-invasive findings raise suspicion of significant shunting or when unexplained ventricular dilation is detected [23,24,30,31,33].

Patients with intermediate-sized CBAFs require closer surveillance, as anatomical size alone does not reliably predict physiological relevance. In this population, clinical follow-up may be performed every 6–12 months. Structural reassessment with TTE is generally recommended on an annual basis to evaluate ventricular dimensions and pulmonary pressures, whereas anatomical imaging with CCTA or CMR angiography is generally reasonable every 2–3 years or earlier when symptoms arise or progressive enlargement is suspected. Functional evaluation assumes greater importance in this subgroup because intermediate fistulas may produce coronary steal despite relatively modest anatomical dimensions. Accordingly, periodic stress perfusion imaging—using SPECT, PET, or stress CMR—may be considered every 1–2 years, particularly in patients with borderline symptoms, progressive enlargement of the fistula, or high levels of physical activity. RHC may be useful at baseline when the haemodynamic significance of the fistula remains uncertain or when non-invasive findings are discordant. Measurement of the shunt fraction (Qp:Qs) allows objective quantification of physiological burden, with values ≥ 1.5 generally considered indicative of haemodynamically significant shunting. Routine serial invasive reassessment is usually unnecessary unless clinical or imaging findings suggest disease progression [23,24,30,31,33].

In contrast, large CBAFs are frequently haemodynamically significant and are associated with a higher risk of myocardial ischaemia, ventricular remodelling, bronchial vascular complications, and aneurysmal degeneration. When definitive closure is temporarily deferred, surveillance should therefore be more intensive. Clinical reassessment may be considered every 6 months, with particular attention to symptoms related to coronary steal or pulmonary complications. TTE should be performed every 6–12 months to evaluate ventricular remodelling and pulmonary pressures, while anatomical reassessment with CCTA or CMR is generally reasonable annually to monitor fistula morphology and detect aneurysmal progression. Because ischaemia is common in this subgroup, stress imaging may be performed on a yearly basis or earlier when symptoms worsen. RHC may be considered at baseline to quantify shunt magnitude and pulmonary artery pressures and may be repeated approximately every 1–2 years if intervention is deferred or when echocardiography suggests increasing pulmonary pressures, progressive ventricular dilation, or clinical deterioration [23,24,30,31,33].

After successful percutaneous or surgical closure, follow-up aims to confirm durable occlusion and detect late complications such as residual shunting, coronary thrombosis, or recanalization. Early post-procedural assessment typically includes clinical evaluation, ECG, and TTE within the first 1–3 months and again at 6 months. Anatomical imaging with CCTA or ICA is often performed within the first year to document complete occlusion and evaluate the morphology of the feeding coronary artery. In patients with previously documented ischaemia or large fistulas, functional testing at 6–12 months may be useful to confirm resolution of coronary steal and establish a new baseline. Thereafter, long-term surveillance generally consists of annual clinical evaluation and echocardiography, with periodic anatomical imaging every 3–5 years when clinically indicated [20,21,31,35,51].

### Long-Term Prognosis

Long-term prognosis in patients with CBAFs is primarily determined by fistula size, physiological significance, and treatment timing. Small asymptomatic fistulas generally carry an excellent prognosis under structured surveillance, whereas intermediate-sized lesions show a more heterogeneous clinical course and therefore require careful longitudinal reassessment. In contrast, large untreated CBAFs are associated with a higher risk of ischaemic, haemodynamic, aneurysmal, and pulmonary adverse events. When closure is performed before irreversible myocardial damage or pulmonary vascular disease develops, both percutaneous and surgical treatments are generally associated with favourable long-term outcomes, durable fistula occlusion, and improvement in symptoms and ischaemic burden [9,21,31,33,35,51].

## 9. Physical Activity and Exercise Recommendations

Physical activity recommendations in patients with CBAFs should be individualised according to fistula size, haemodynamic significance, and the presence or absence of inducible myocardial ischaemia. Although robust prospective data are lacking, contemporary recommendations are largely extrapolated from congenital heart disease and sports cardiology guidelines [32,33,55].

In small CBAFs without inducible ischaemia, ventricular dysfunction, or pulmonary hypertension, participation in recreational and moderate-intensity physical activity is generally considered safe. Competitive sports may also be allowed after exclusion of ischaemia through functional testing in highly active individuals. Periodic reassessment with clinical evaluation and imaging is advisable, particularly in younger patients and athletes [55].

In intermediate-sized CBAFs, exercise recommendations should be guided by functional assessment. When stress imaging demonstrates absence of inducible ischaemia and haemodynamic evaluation confirms the absence of significant shunting (Qp:Qs < 1.5), low- to moderate-intensity exercise is usually permissible. However, participation in high-intensity competitive sports should generally be restricted unless comprehensive functional evaluation—including stress perfusion imaging and, when appropriate, haemodynamic assessment—excludes ischaemia or significant coronary steal [55].

In large or haemodynamically significant CBAFs, high-intensity exercise and competitive sports are generally discouraged prior to definitive treatment because of the potential risk of ischaemia, arrhythmias, or aneurysmal complications. Only light recreational activity may be permitted until closure of the fistula has been achieved and follow-up functional testing confirms resolution of ischaemia [55].

Following successful closure of a fistula, gradual return to physical activity is usually possible once complete occlusion and absence of residual ischaemia have been confirmed [20,55].

Exercise recommendations according to fistula size and physiological significance are summarised in Table 2.

## 10. Conclusions and Future Perspectives

CBAFs represent a rare but clinically relevant subset of coronary vascular anomalies. The increasing use of multimodality cardiovascular imaging—particularly CCTA—has markedly improved the detection and anatomical characterisation of these lesions, leading to growing recognition in contemporary clinical practice [29,30,31,33,37].

Although many CBAFs remain asymptomatic and are discovered incidentally, others may lead to clinically important complications, including myocardial ischaemia related to coronary steal physiology, ventricular remodelling, arrhythmias, haemoptysis, and aneurysmal degeneration of the fistulous tract. The clinical impact of these anomalies is largely determined by fistula size, shunt magnitude, and the presence of associated cardiovascular or pulmonary disease [11,18,31,33].

Optimal management therefore requires an integrated approach combining detailed anatomical imaging, functional ischaemia testing, and—in selected cases—invasive haemodynamic assessment. Conservative surveillance is appropriate for small asymptomatic fistulas, whereas percutaneous or surgical closure is generally recommended for symptomatic or haemodynamically significant lesions. Both treatment strategies have demonstrated favourable long-term outcomes when applied in appropriately selected patients [21,26,27,31,33].

The practical size-based framework summarised in Table 2 may assist clinicians in translating heterogeneous anatomical and physiological findings into individualised management and surveillance strategies, while Figure 2 and Figure 3 provide complementary procedural and longitudinal decision pathways [21,24,31,33].

Several areas remain insufficiently explored. Prospective multicentre registries are needed to better define the natural history of CBAFs and to refine risk stratification across different patient subsets. Standardisation of size-based classification systems and surveillance strategies would help harmonise clinical practice. In addition, advances in computational flow modelling and quantitative perfusion imaging may improve assessment of fistula-related ischaemia and guide individualised therapeutic decisions [15,30,31,47,49].

Ultimately, improved understanding of the pathophysiology, natural history, and optimal management of CBAFs will require collaborative research efforts integrating imaging, interventional cardiology, and congenital heart disease expertise [15,30,31].

## Figures and Tables

**Table 1:** Strengths and limitations of the principal multimodality imaging modalities used in the diagnostic evaluation of coronary–bronchial artery fistulas.

Imaging Modality	Strengths	Limitations
**CCTA**	First-line for anatomical definition Excellent 3D delineation of origin, course, drainage, and aneurysmal segments Defines mediastinal/pulmonary relationships Simultaneous CAD assessment	Limited physiological assessment Radiation and iodinated contrast Cannot reliably distinguish steal from flow-limiting CAD without functional testing
**ICA**	Real-time coronary and fistula flow visualisation Essential for transcatheter planning Can be combined with invasive coronary physiology	Two-dimensional and projection-dependent Limited for complex extracardiac anatomy Invasive; radiation/contrast exposure
**TTE/TEE**	Widely available and radiation-free Assesses chamber size, ventricular function, valvular disease, and pulmonary pressures Useful for baseline assessment and follow-up	Limited direct visualisation of the fistula in adults Inferior anatomical definition compared with CCTA/CMR
**SPECT/PET**	Functional ischaemia assessment Detects steal-related perfusion defects Useful when anatomical size does not predict significance	No detailed anatomical characterisation Radiation exposure (especially SPECT) Often requires correlation with CCTA/ICA when CAD coexists
**CMR/stress CMR**	Radiation-free multiparametric assessment Evaluates ventricular volumes, function, stress perfusion, and fibrosis Detects ischaemia, scar, and biventricular remodelling	Lower spatial resolution than CCTA for distal/tortuous fistulas Limited coronary–bronchial anatomical mapping in small/complex lesions Lower availability/longer acquisition time
**RHC**	Invasive haemodynamic quantification Measures Qp:Qs, pulmonary pressures, and pulmonary vascular resistance Useful when haemodynamic significance remains uncertain	No anatomical delineation of the fistula Invasive Not routinely required in all patients

Abbreviations: CAD = coronary artery disease; CCTA = coronary computed tomography angiography; CMR = cardiac magnetic resonance; ICA = invasive coronary angiography; PET = positron emission tomography; RHC = right heart catheterization; SPECT = single-photon emission computed tomography; TEE = transesophageal echocardiography; TTE = transthoracic echocardiography.

**Table 2:** Size-based clinical management and surveillance strategy for coronary–bronchial artery fistulas.

	Small CBAFs	Intermediate CBAFs	Large CBAFs
**Physiological significance**	Minimal shunt; coronary steal uncommon	Variable; physiological relevance cannot be inferred from size alone	Usually haemodynamically significant; higher risk of steal, remodelling, and aneurysmal change
**Typical presentation**	Usually incidental and asymptomatic	Variable; angina, dyspnoea, or haemoptysis may occur	Often symptomatic, with ischaemia, dyspnoea, haemoptysis, or ventricular remodelling
**Clinical follow-up**	Every 12–24 months	Every 6–12 months	Every 6 months
**TTE**	Every 2–3 years	Yearly, or earlier if clinical status changes	Every 6–12 months
**CCTA/CMR**	Every 3–5 years	Every 2–3 years, or earlier if progression is suspected	Yearly if closure is deferred
**Stress imaging**	Not routine; symptom-driven	Every 1–2 years if symptoms, high physical activity, or borderline physiology	Every 1–2 years, or earlier if symptoms worsen
**RHC**	Not routine; consider if chamber dilatation or significant shunt is suspected	Baseline if physiological significance remains uncertain; repeat if progression is suspected	Baseline to quantify Qp:Qs and pulmonary pressures; repeat if progression is suspected
**Coexisting CAD**	Treat CAD according to guidelines; close the fistula only if steal-related ischaemia is demonstrated	Integrate CAD burden and steal physiology; individualised strategy	PCI or CABG as indicated, together with fistula closure
**Physical activity**	Recreational and moderate exercise; competitive sport only if ischaemia is excluded	Low-to-moderate exercise if no ischaemia or significant shunt is present; competitive sport usually discouraged	Avoid high-intensity or competitive sport until closure; light recreational activity only
**When to close**	Rare; consider closure if ischaemia, haemoptysis, aneurysmal enlargement, or progressive dilatation occurs	Consider closure if symptoms, ischaemia, Qp:Qs ≥ 1.5, or progressive dilatation are present	Closure generally recommended
**Preferred approach**	Conservative surveillance	Individualised, physiology-guided management	Closure strategy based on anatomical suitability and surgical complexity

Abbreviations: CABG = coronary artery bypass grafting; CAD = coronary artery disease; CBAFs = coronary–bronchial artery fistulas; CCTA = coronary computed tomography angiography; CMR = cardiac magnetic resonance; PCI = percutaneous coronary intervention; Qp:Qs = pulmonary-to-systemic flow ratio; RHC = right heart catheterization; TTE = transthoracic echocardiography.

## Data Availability

No new data were created or analysed in this study.
